# Evolutionary Multi-Objective One-Shot Filter Pruning for Designing Lightweight Convolutional Neural Network

**DOI:** 10.3390/s21175901

**Published:** 2021-09-02

**Authors:** Tao Wu, Jiao Shi, Deyun Zhou, Xiaolong Zheng, Na Li

**Affiliations:** School of Electronics and Information, Northwestern Polytechnical University, 127 West Youyi Road, Xi’an 710072, China; tao_woe@mail.nwpu.edu.cn (T.W.); dyzhounpu@nwpu.edu.cn (D.Z.); xlzheng@mail.nwpu.edu.cn (X.Z.); linaflydream@mail.nwpu.edu.cn (N.L.)

**Keywords:** convolutional neural network, filter pruning, evolutionary multi-objective algorithm, lightweight model

## Abstract

Deep neural networks have achieved significant development and wide applications for their amazing performance. However, their complex structure, high computation and storage resource limit their applications in mobile or embedding devices such as sensor platforms. Neural network pruning is an efficient way to design a lightweight model from a well-trained complex deep neural network. In this paper, we propose an evolutionary multi-objective one-shot filter pruning method for designing a lightweight convolutional neural network. Firstly, unlike some famous iterative pruning methods, a one-shot pruning framework only needs to perform filter pruning and model fine-tuning once. Moreover, we built a constraint multi-objective filter pruning problem in which two objectives represent the filter pruning ratio and the accuracy of the pruned convolutional neural network, respectively. A non-dominated sorting-based evolutionary multi-objective algorithm was used to solve the filter pruning problem, and it provides a set of Pareto solutions which consists of a series of different trade-off pruned models. Finally, some models are uniformly selected from the set of Pareto solutions to be fine-tuned as the output of our method. The effectiveness of our method was demonstrated in experimental studies on four designed models, LeNet and AlexNet. Our method can prune over 85%, 82%, 75%, 65%, 91% and 68% filters with little accuracy loss on four designed models, LeNet and AlexNet, respectively.

## 1. Introduction

Recently, deep neural networks have achieved significant development with the innovations of computing equipment, especially for GPU-based computing. The excellent performance of deep neural networks has led to their applications in many fields such as computer vision, speech recognition and natural language processing [1]. For practical applications, convolutional neural networks are more widely used than fully connected models, because convolutional kernels can extract more potential spatial features with less weight parameters. Nowadays, convolutional neural networks are not only widely used for image recognition [2,3,4], but also achieve perfect performance in natural language processing [5,6,7]. However, better performance always means more complex model structures [8], which limit the more practical applications of deep neural networks. For example, sensor platforms always suffer from limited computation and storage resources, and it is hard to perform deep models on them. Therefore, designing a lightweight neural network with high performance is necessary and valuable for performing them on resource-limited platforms. Moreover, edge computing is widely used in practical applications such as intelligent city, and these edge devices are always resource-limited. For these edge devices, although sometimes they handle the same problems, their requirements for results may be different because of different environments and equipment hardware limitations. Therefore, we may face a challenge in designing a series of similar models for these edge devices. Neural network pruning is an efficient method to simplify network structure and maintain the performance of the original complex model. Therefore, in this paper, we will study how to design a lightweight convolutional neural network based on pruning methods that can be deployed on resource-limited devices.

Factually, studies [8,9] have shown that neural networks always have redundant parameters. Therefore, removing some redundant or unimportant parameters can simplify neural network structure with little or even no loss of accuracy. Neural network pruning is a traditional technique, which can be divided into unstructured pruning (e.g., weights pruning) and structured pruning (e.g., filter pruning) [10,11]. Unstructured pruning methods prune individual parameters. Doing so produces a sparse neural network, which—although smaller in terms of parameter-count—may not be arranged in a fashion conducive to speed-ups using modern libraries and hardware [11,12]. Structured pruning methods consider parameters in groups, removing entire neurons, filters or channels to exploit hardware and software optimized for dense computation [13,14,15,16,17]. The neural network pruning method can be divided into iteration pruning and one-shot pruning from another point of view. Iterative neural network pruning methods [18,19,20,21] repeat pruning and fine-tuning operations while one-shot pruning methods [22,23,24] run pruning and fine-tuning operations only one time. The lottery ticket hypothesis [24] indicated that one-shot pruning makes it possible to identify winning tickets without repeated training, especially for the small size networks where the winning ticket is the sub-networks which reach test accuracy comparable to the original network. Whatever neural network pruning method is used, neuron or connection importance estimation is key to these methods. The literature [25,26] has studied how to understand the importance of individual parameters or parameters in the group.

We still face some issues for convolutional network pruning. Firstly, the unstructured pruning methods can obtain a sparse model, but it is also hard to be deployed on resource-limited devices without specific sparse computing algorithms. For structured pruning methods, the key problem is how to distinguish which group of parameters can be removed. Although some studies have proposed different metrics of importance estimation, there is still no uniform and comprehensive pruning standard. Secondly, handcraft technology-based pruning methods may not select optimal pruning parameters. Moreover, it is hard to balance the model scale and model performance, or we will incur significant time and resource costs to obtain a good trade-off between the model scale and model performance. Thirdly, compared with one-shot pruning, although iterative pruning methods are can obtain better results with more ease, iterative pruning methods always have larger computation complexity and would cost more time. The time cost of a human is very expensive for iterative pruning methods, especially for designing a series of similar models. Whether it is iterative pruning or one-shot pruning, we need to design each model independently for each device.

In this paper, we propose an evolutionary multi-objective one-shot filter pruning method (EMOFP) for designing a lightweight convolutional neural network. In EMOFP, there are three phases: obtaining a well-trained CNN; evolutionary multi-objective filter pruning (EMFP); and a fine-tuning pruned model; these phases are implemented only one time. It is important to note that the key of the EMOFP is the second phase. In EMFP, we build a constraint multi-objective filter pruning problem, in which two objective functions represent the pruning ratio and the accuracy of the pruned model, respectively. Then, a non-dominated sorting-based evolutionary multi-objective algorithm is used to solve the above filter pruning problem. Finally, for a series of different trade-off pruning solutions of EMFP, we uniformly select some pruned model to the third phase, fine-tuning the selected pruned models. In total, our EMOFP automatically completes the filter pruning task without extra human hand-craft and pruning and fine-tuning only once, and provides a series of pruned models with a different trade-off between the model scale and model performance.

The remainder of this paper is organized as follows. In Section 2, we will review the context of the proposed method and some related works. In Section 3, the evolutionary multi-objective one-shot filter pruning method will be introduced in detail. We will conduct the experimental studies in Section 4. A discussion about one-shot and iterative neural network pruning will be introduced in Section 5. Finally, we will give the concluding remarks of this paper.

## 2. Background and Related Work

### 2.1. Neural Network Pruning

According to the literature [11,12], we define a neural network architecture as a function family f(X;·), where *X* denotes the dataset on which to train and fine-tune. The architecture of a neural network includes the configuration of a network’s parameters and the set of operations which consists of convolutional units, activation functions, pooling, batch normalization, etc. Therefore, we can define a neural network model as the particular parameterization of an architecture. For example, for a specific parameter *W*, the model can be denoted as f(X;W). The neural network pruning aims to obtain a new $W^{\prime }$ which is simpler than the original *W*. Generally, we can obtain $W^{\prime }$ by

(1) $$\begin{array}{c} W^{\prime }=M⊙W\\ M\in {\left\{0,1\right\}}^{\left|W\right|} \end{array}$$

where M denotes the binary pruning mask in which zero means the parameter will be pruned, ⊙ is the Hadamard product operator and |W| means the number of elements in *W*. In practice, rather than using an explicit mask, the pruned parameters of *W* are fixed to zero or removed entirely.

A general framework of neural network pruning can be summarized in Algorithm 1. The neural network pruning task can be divided into three phases [12]. In the first phase, we trained a dense neural network model on the dataset *X* to obtain the original complex model. In the second phase, we prune the original complex model with some different pruning strategies to obtain the pruned model. In the third phase, in order to maintain the accuracy of the original model, we fine-tuned the pruned model on the dataset *X*. Furthermore, for iterative pruning methods, the second and third phases will be executed alternately for *N* iterations. When *N* is equal to 1, this framework represents a one-shot neural network pruning method.
**Algorithm 1:** General Framework of Pruning and Fine-Tuning
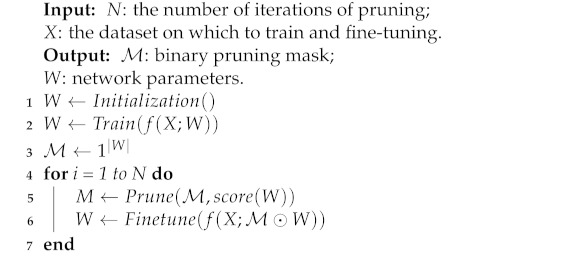


Most neural network pruning methods focus on proposing or improving pruning strategies in the second phase. For example, an early approach to pruning was biased weight decay which is based on weight magnitude [27]. The optimal brain damage (OBD) [18] and optimal brain surgeon (OBS) [19] reduced the number of connections based on the Hessian of the loss function. In the literature [13,28], a structured sparsity regularizer was added on each layer to reduce trivial filters, channels or even layers. These works used the $l_{1}$ or $l_{2}$-norm to select and prune unimportant filters, channels, etc.

### 2.2. Evolutionary Multi-Objective Optimization

Multi-objective optimization is used to solve multi-objective optimization problems (MOPs), which consist of more than one objective function and all of the objective function needs to be optimized simultaneously. Therefore, an MOP can be stated as

(2) $$\begin{array}{cc} \min_{x}\; & F\left(x\right)={\left(f_{1}\left(x\right),f_{2}\left(x\right),\ldots ,f_{m}\left(x\right)\right)}^{T},\\ s.t.\; & x={\left(x_{1},x_{2},\ldots ,x_{n}\right)}^{T} \end{array}$$

where *m* means the number of objective functions, and *n* denotes the dimension of decision variable. In general, the objectives in MOP are in conflict with each other. Therefore, for solution $x_{a}$ and $x_{b}$, if and only if the following conditions are satisfied:

(3) $$\begin{array}{cc} \; & \forall i=1,2,\cdots ,m\;f_{i}\left(x_{a}\right)\leq f_{i}\left(x_{b}\right),\\ \; & \exists j=1,2,\cdots ,m\;f_{j}\left(x_{a}\right)<f_{j}\left(x_{b}\right), \end{array}$$

then solution $x_{a}$ is better than solution $x_{b}$. It is generally called $x_{a}$ dominate $x_{b}$, and it is always marked as $x_{a}≻x_{b}$. Moreover, the Pareto optimal solution means the solution which cannot be dominated by any other solutions in decision space. All Pareto solutions compose the Pareto optimal set, and the Pareto front is the set of all Pareto optimal objective vectors corresponding to the Pareto optimal set.

Generally, it is very difficult to obtain whole Pareto optimal solutions. Therefore, multi-objective optimization needs to obtain a uniformly distributed Pareto front which can represent the whole solutions approximately. Nowadays, most multi-objective algorithms are designed based on population optimization, especially with evolutionary algorithms, such as the non-dominated sorting genetic algorithm (NSGA-II) [29], multi-objective particle swarm optimization (MOPSO) [30,31] and multi-objective evolutionary algorithm based on decomposition (MOEA/D) [32]. In this paper, we will use NSGA-II to solve the multi-objective filter pruning problem, which is a combinatorial optimization method. MOPSO and MOEA/D are more efficient for continuous optimization problems. The key technologies of NSGA-II are fast non-dominated sorting and crowded-distance-based selection, which provide Pareto solutions and make them uniformly distributed, respectively.

Multi-objective optimization has been widely used to solve machine learning problems. For example, using multi-objective to sparse reconstruction was proposed in [33]. In [34,35], multi-objective optimization was used for deep learning. Self-pace learning can also be combined with multi-objective optimization [36]. Furthermore, a multi-objective matrix decomposition method is proposed in [37]. For neural network structure optimization, Lu et al. have proposed NSGA-Net [38] which considers the model computational cost and accuracy as an MOP and solves it with NSGA-II. A continuous evolution for an efficient neural architecture search was proposed in [39]. Moreover, we proposed a multi-objective particle swarm optimization for neural network pruning [40], in which the pruning ratio of each layer is optimized with two objectives of global pruning ratio and pruned model’s accuracy. In summary, multi-objective optimization is an efficient method to solve neural network optimization problems, especially if it is well worked on neural network pruning.

## 3. Methodology

In this section, we introduce the proposed EMOFP in detail. Firstly, the overview framework of multi-objective one-shot filter pruning is presented. Then, we introduce the mathematical model of filter pruning, especially the two conflicting objective functions. Moreover, an evolutionary multi-objective algorithm was used to prune the filters of the convolutional neural network in detail. Finally, we fine-tune the pruned model to maintain the accuracy as much as possible.

### 3.1. Framework of EMOFP

**Figure 1:**
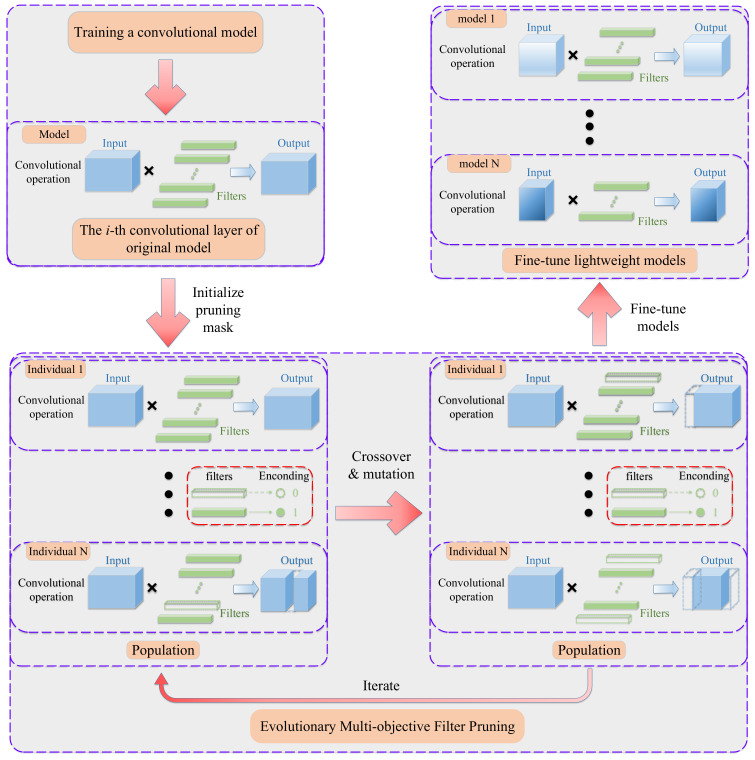
The framework of evolutionary multi-objective one-shot filter pruning.

In our EMOFP framework, the main works can be divided into three phases. Firstly, we obtain an original well-trained convolutional model, which may be trained by ourselves or is a public well-trained model. Secondly, we prune the original model by evolutionary multi-objective filter pruning method, and obtain a series of trade-off pruned models. Lastly, models from the second phase would be fine-tuned to improve the performances of these models. Therefore, our method outputs a series of different trade-off lightweight convolutional models. A detailed framework is shown in Figure 1. In the framework of EMOFP, the main work of our proposed method is in the second phase, evolutionary multi-objective filter pruning. Figure 1 also shows an illustration of filter pruning for the convolutional model, in which the *i*-th convolutional layer can be represented as the product of the input tensor and filters. Taking a convolutional layer as an example, in the second phase, we initialize a population which consists of *N* different filter pruning schemes. The virtual filter represents the filter that will be removed and the solid filter indicates the filter that will be retained in Figure 1, and we use 0 and 1 to encode the removed and retained filter, respectively. Moreover, we used evolutionary operators (cross-over and mutation) to update the population and finally output a Pareto front which consists of a series of different trade-off filter pruning schemes.

### 3.2. Multi-Objective Filter Pruning Model

In order to parameterize the filter pruning for convolutional neural networks, we assume that *W* denotes the filters of a convolutional model in which w∈W means a complete filter, and f(X;W) denotes the model in which *X* means the dataset used to train and fine-tune. Moreover, the acc, which is the accuracy of the models, is used to evaluate the performance of models. The pruning operation can be represented as the Hadamard product of filters *W* and pruning binary mask M, in which the filter will be turned on/off when the corresponding mask equals 1/0. We can thus present the filter pruning of convolutional neural networks as in Equation (1), and the pruned model is f(X;M⊙W).

In the filter pruning task, we not only need to obtain as simple neural networks as possible, but we also need to retain the performance of obtained neural networks as much as possible. Therefore, we designed the two following objective functions:

(4) $$\left\{\begin{array}{cc} obj_{1}\left(M\right) & ={∥M∥}_{l_{0}}\\ obj_{2}\left(M\right) & =|acc(f(X;W))-acc(f(X;M⊙W))| \end{array}\right.$$

where the first objective function means the number of non-zero elements in the pruning mask M which can also denote the number of retained filters, and the second objective function represents the performance difference between original and pruned models. Finally, we can establish a multi-objective filter pruning model as

(5) $$\begin{array}{cc} \min_{M}\;F\left(M\right) & ={\left(obj_{1}\left(M\right),obj_{2}\left(\left(M\right)\right)\right)}^{T}\\ \; & ={\left({∥M∥}_{l_{0}},\left|acc\left(f\left(X;W\right)\right)-acc\left(f\left(X;M⊙W\right)\right)\right|\right)}^{T} \end{array}$$

The numerical range of a first objective function ${∥M∥}_{l_{0}}$ is a positive integer, while the value of the second objective function is in the range [0, 1]. A large numerical difference between these two objectives may cause unbalanced solutions and the first objective may lead the optimization. Therefore, we need to normalize the first objective to prevent this issue, and the normalized multi-objective filter pruning model is:

(6) $$\min_{M}\;F\left(M\right)={\left(\frac{{∥M∥}_{l_{0}}}{\left|M\right|},\left|acc\left(f\left(X;W\right)\right)-acc\left(f\left(X;M⊙W\right)\right)\right|\right)}^{T}$$

where |M| means the number of elements in M. Furthermore, the accuracy of the original model is a constant, and we cannot fine-tune a very bad pruned model to obtain a performance similar to that of the original model. Therefore, it is necessary to add constraints about the accuracy of the pruned model before fine-tuning. Thus, the final multi-objective filter model can be represented as

(7) $$\begin{array}{cc} \min_{M}\;F\left(M\right) & ={\left(\frac{{∥M∥}_{l_{0}}}{\left|M\right|},\left|acc\left(f\left(X;W\right)\right)-acc\left(f\left(X;M⊙W\right)\right)\right|\right)}^{T}\\ \; & ={\left(\frac{{∥M∥}_{l_{0}}}{\left|M\right|},\left|C-acc\left(f\left(X;M⊙W\right)\right)\right|\right)}^{T}\\ \; & ={\left(\frac{{∥M∥}_{l_{0}}}{\left|M\right|},1-acc\left(f\left(X;M⊙W\right)\right)\right)}^{T}\\ s.t.\; & \delta_{1}\leq 1-acc\left(f\left(X;M⊙W\right)\right)\leq \delta_{2} \end{array}$$

where *C* denotes a constant, and $\delta_{1}$ and $\delta_{2}$ are error constraints of the pruned model, designating the acceptable error range of pruned model. For the second objective function, we finally use 1−acc(f(X;M⊙W)), which means the error of the models.

### 3.3. Evolutionary Multi-Objective Filter Pruning Algorithm

For the above multi-objective filter pruning model, we use an evolutionary multi-objective filter pruning (EMFP) algorithm based on NSGA-II to optimize it. The pseudocode of EMFP is shown in Algorithm 2. In algorithm EMFP, we binarily encode the pruning masks as individuals of an evolutionary population, and genetic operations, such as crossover and mutation, are used to generate offspring, whilst finally the nondominated sorting and crowding distance-based selection are applied to update the population and Pareto front. We will then introduce some detailed operations in EMFP.
**Algorithm 2:** Pseudocode of evolutionary multi-objective filter pruning
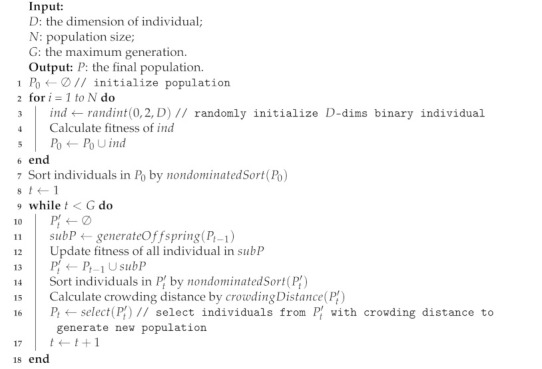


**Figure 2:**
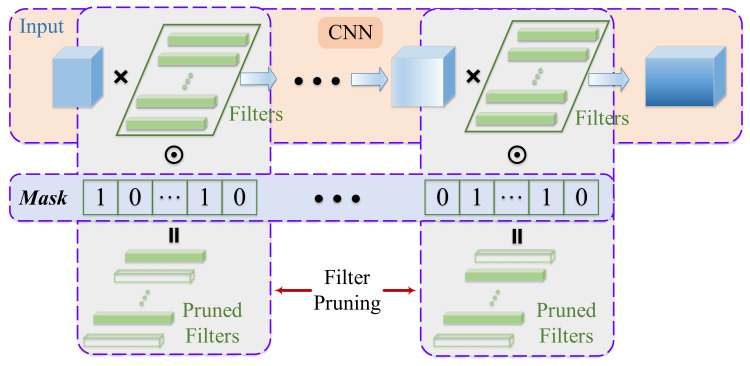
Illustration of filter pruning and evolutionary individual encoding.

Firstly, we introduce individual encoding and population initialization. Figure 2 gives a simple illustration about filter pruning and evolutionary individual encoding. For each convolutional layer in the CNN model, we generate a binary mask with a size equal to the number of filters, and each element in the mask represents the pruning decision about the corresponding filter, 0 means that the filter needs to be removed, and 1 indicates the remaining filter. For example, the mask ${\left(1,0,\ldots ,1,0\right)}^{T}$ means that the second and last filter need to be removed. We concatenated all the masks of each convolutional layer to obtain the total filter pruning mask for the CNN model, such as the Mask in Figure 2. In EMFP, the decision variable is the pruning mask, so we encode the concatenated mask vector as the individual. Decoding needs to decompose the concatenated mask into the masks of each convolutional layer according to the model configuration. For example, for a CNN model with two layers, the number of filters of each layer is 4 and 6, respectively. Therefore, the configuration of the CNN model is [4, 6]. A reasonable encoding is ${\left(1,0,1,0,0,0,1,0,1,0\right)}^{T}$, which can be decoded to two sub-masks ${\left(1,0,1,0\right)}^{T}$ and ${\left(0,0,1,0,1,0\right)}^{T}$ for two convolutional layers, respectively. The population initialization is shown in lines 1–6 in Algorithm 2. We randomly initialize the binary individual and repeat *N* times to generate the population, and the fitness calculation is based on Equation (7).

Secondly, we simply introduce generating offspring in line 11 in Algorithm 2. In order to generate new individuals, we firstly randomly select two individuals—$ind_{1}$ and $ind_{2}$—from the current population $P_{t}$. Furthermore, we will then use a binary two-point crossover Equation (8) to create a new individual $ind_{c}$ when the probability of crossover is satisfied. Then, if the probability of mutation is satisfied, we will use the bitflip mutation Equation (9) to mutate the individual $ind_{c}$ to obtain the mutated individual $ind_{m}$. Finally, we repeat the above operations *N* times to generate the offspring population subP:

(8) $$ind_{c}\left(i\right)=\left\{\begin{array}{cc} ind_{1}\left(i\right)\; & \mathrm{if}\;cp_{1}\leq i\leq cp_{2}\\ ind_{2}\left(i\right)\; & \mathrm{otherwise} \end{array}\right.$$

where $cp_{1}$ and $cp_{2}$ are two randomly selected crossover points:

(9) $$ind_{m}\left(i\right)=\left\{\begin{array}{cc} \neg ind_{c}\left(i\right)\; & \mathrm{if}\;rand\leq pm\\ ind_{c}\left(i\right)\; & \mathrm{otherwise} \end{array}\right.$$

where pm means the probability of mutation. The derailed descriptions about the nondominated sort and crowding distance can refer to the literature [29].

### 3.4. Fine-Tuning Strategy

After the above EMFP, we obtain a series of lightweight models. These models need to be trained again, because missing a large number of parameters results in their performance’s degradation. Compared with absolutely retraining the pruned lightweight model with randomly initialized weight parameters, fine-tuning is a more efficient method to recover the performance of models [24]. Therefore, in the EMOFP framework, we use a fine-tuning strategy to improve the performance of valuable pruned models after an evolutionary filter pruning operation.

In the model fine-tuning phase, we need to select some valuable pruned model before fine-tuning the lightweight neural networks, because the EMFP algorithm outputs a series of trade-off pruned models. For selecting suitable pruned models, we followed the rule of uniform selection. For example, if we need to select *K* models, we will sort all models according to the number of filters (the first objective in the multi-objective filter pruning model), and then uniformly select *K* models from the model set. For selected pruned models, we fine-tune them with the strategies of the data augmentation and auto-reduced learning ratio.

### 3.5. Computational Complexity of EMOFP

Before analyzing the computational complexity of the proposed EMOFP, we assumed some computational costs of special operators. Assuming that the computational cost of the neural network training is O(T), and the complexity of fitness evaluation is O(F), both consist of the filter pruning and model evaluation on test data. The computation complexity of EMOFP can be divided into two parts: the computational cost of the EMFP algorithm (Algorithm 2) and the computational cost of the fine-tuning pruned model. For the first part, the population size and maximum generation are *N* and *G*, respectively. The computational complexity of initialization, the nondominated sort and calculating the crowding distance are O(NF), $O(2N^{2})$ and O(2(N−2)), respectively. Therefore, the total computational complexity of Algorithm 2 is $O(NF+2N^{2}+G\left(2N+NF+2N^{2}+2\left(N-2\right)+N\right))$, which can be denoted by $O(GN^{2}+GNF)$ in summary. If *K* stands for the number of pruned models selected for fine-tuning, the computational complexity of the fine-tuning phase is O(KT). Thus, the total computational complexity of our proposed EMOFP is $O(GN^{2}+GNF+KT)$. It is noted that the computation complexity in this part means the time complexity, and it is based on the assumed time cost of the neural network training O(T) and individual fitness evaluation O(F).

## 4. Experimental Studies

In this section, we demonstrate the performance of the proposed EMOFP with experimental studies. Firstly, we introduce the experimental setting and related convolutional neural networks and datasets. Secondly, the overall experimental results—especially the comparison results—are shown. Lastly, we present and analyze the experimental results of our EMOFP on these models in detail.

### 4.1. Description of Model Variants and Datasets

In our experimental studies, we designed four convolutional models which have a different number of convolutional layer and the same fully connected layers. Moreover, we also apply our EMOFP on common yet efficient models, LeNet and AlexNet. Simple descriptions of used neural network models are shown as follow.

- Conv1: Conv1 is a simple convolutional neural network which consists of a convolutional layer and two fully connected layers. In the convolutional layer, there are 64 filters with the size of 3×3, and the number of neurons in fully connected layers are 128 and 10. The dataset for Conv1 is MNIST.
- Conv2: Conv2 is a convolutional neural network which consists of two convolutional layers and two fully connected layers. The first convolutional layer has 32 filters with the size of 3×3 while the second convolutional layer with 64 3×3 filters, and the number of neurons in fully connected layers are 128 and 10. The dataset for Conv2 is MNIST.
- Conv3: Conv3 is a convolutional neural network which consists of three convolutional layers and two fully connected layers. The number of filters of each layer are 16, 32 and 64, respectively; the filter size of all filters is 3×3, and the number of neurons in fully connected layers are 128 and 10. The dataset for Conv3 is MNIST.
- Conv4: Conv4 is a convolutional neural network which consists of four convolutional layers and two fully connected layers. The number of filters of each layer is 16, 32, 64 and 64, respectively; the filter size of all filters is 3×3, and the number of neurons in the fully connected layers are 128 and 10. The dataset for Conv4 is MNIST;
- LeNet: LeNet [41] is a classical and common convolutional neural network for MNIST classification. LeNet has two convolutional layers and three fully connected layers. The number of filters of two convolutional layers is 8 and 16, and the size of filter is 5×5 for each layer. The number of three connected layers is 120, 84 and 10.
- AlexNet: AlexNet [2] is a classical deep convolutional neural network which was designed for ImageNet Large Scale Visual Recognition Challenge (ILSVRC) in 2012. AlexNet has five convolutional layers and three fully connected layers. In our experiment, we properly simplified AlexNet to classify CIFAR10.

Detailed model variants, such as configurations, accuracy and used datasets are presented in Table 1. MNIST has a training set of 60,000 examples and a test set of 10,000 examples of handwritten digits. The images are centered in a 28×28 image. CIFAR10 consists of 60,000 color images in 10 classes with the size of 32×32, and each class has 6000 images. There are 50,000 training images and 10,000 test images in CIFAR10.

### 4.2. Experimental Setting

Firstly, we define some evaluation metrics to measure the pruned model, which consist of the error, relative error, filter compression ratio and floating point operations (FLOPs) of the pruned model. A detailed description of these is shown as follows.

- Error: The error of the neural network by
  (10) $$\mathrm{Error}=\frac{FP+FN}{TP+FP+TN+FN},$$
  where TP—true positive—is the number of observations correctly assigned to the positive class; TN—true negative—is the number of observations correctly assigned to the negative class; FP—false positive—is the number of observations assigned by the model to the positive class which in reality belong to the negative class; and FN—false negative—is the number of observations assigned by the model to the negative class which actually belong to the positive class.
- Relative error (RE): We define relative error of pruned model as
  (11) $$\mathrm{RE}=\frac{E_{p}-E_{o}}{E_{o}}\times 100\%$$
  where $E_{p}$ and $E_{o}$ denote the error of the pruned model and original model, respectively. A positive RE means that the error of pruned model is worse than the original error, and a negative RE means the better performance of pruned model.
- Compression ratio (CR): We define the compression ratio of filter pruning as
  (12) $$\mathrm{CR}=\frac{N_{o}}{N_{p}}$$
  where $N_{p}$ and $N_{o}$ denote the filter’s number of pruned models and original models, respectively.
- Floating point operations (FLOPs): FLOPs can effectively measure a model’s computation resources, and it is always used to measure model complexity in practical applications.

For exploring filter pruning effectiveness, all experiments simply remove the whole filter with fixed fully connected layers. In our experiments, we use the pymoo [42] package to perform evolutionary multi-objective optimization with NSGA-II, and all operations about convolutional neural networks are based on Keras [43]. The detailed parameter settings of our EMOFP are shown in Table 2, where the lower and upper bounds of the error in Equation (7) are two parameter sets. For our designed models and LeNet, the $\delta_{1}$ and $\delta_{2}$ are 0.01 and 0.7, respectively. Furthermore, the error bounds of the pruned model is 0.5 and 0.9 when we prune AlexNet, because AlexNet has a much larger number of weights and redundant weights for classifying CIFAR10.

The comparison methods used in this paper are the $l_{1}$-norm and $l_{2}$-norm-based filter pruning [13]. The difference between these two filter pruning methods is the different filter importance estimation with the $l_{1}$-norm and $l_{2}$-norm. After estimating the importance of all filters, we can remove unimportant filters with a given pruning ratio. Moreover, there are two different pruning ratio assignment schemes: the global pruning ratio assignment and the layer-wise pruning ratio assignment. Therefore, the comparison methods have four different types which can be recorded as $l_{1}$-global, $l_{1}$-layer, $l_{2}$-global and finally as $l_{2}$-layer.

Because EMOFP is a one-shot pruning method, the comparison methods will also use a one-shot pruning framework for fair comparison. Moreover, EMOFP provides a series of trade-off solutions, and comparison methods only obtain a pruning result in one running. Therefore, in order to better compare, we select three different trade-off solutions which are located in the head, middle and bottom of the Pareto front, respectively. Then, the comparison methods pruned the model with same pruning ratio with selected solutions. Finally, for each model, we provide three different results with a different pruning ratio for each method.

### 4.3. Experimental Results Presentation and Analysis

#### 4.3.1. Results on Designed Models

**Figure 3:**
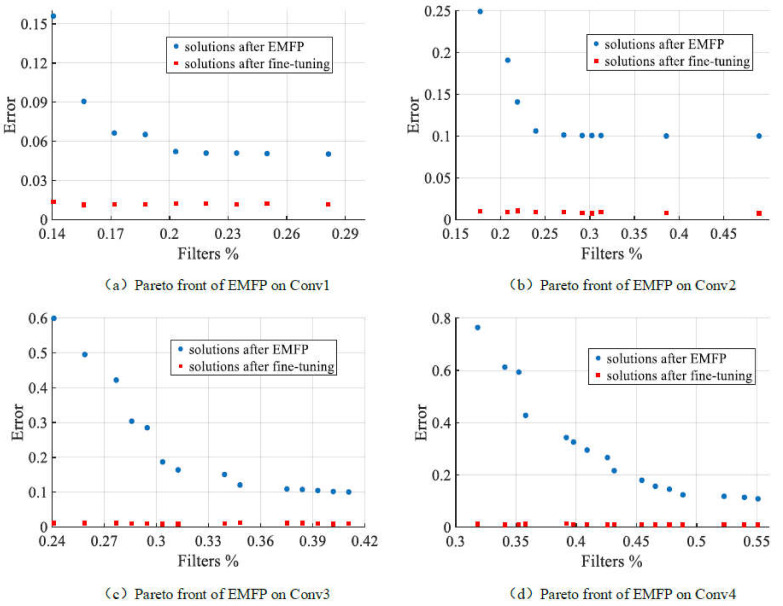
Pareto fronts of EMFP and scatter plots of fine-tuned solutions on designed models. Subfigure (**a**–**d**) mean the scatter plots on Conv1, Conv2, Conv3 and Conv4, respectively.

In this part, we present the experimental results of our designed models. The pruning results on Conv1, Conv2, Conv3 and Conv4 are shown in Table 3, Table 4, Table 5 and Table 6, respectively. Moreover, we plot the Pareto fronts of EMFP on four designed models in Figure 3.

First of all, from Figure 3a, it is well known that we can obtain a series of trade-off pruned models after running EMFP, and the remained filter ratio is in the range of [0.14 0.29] while most of them have an error which is less than 0.1. Compared results with other methods are presented in Table 3, EMOFP obtains better pruned models, especially before fine-tuning. From Table 3, the configurations of pruned models are 18, 13 and 9 for all pruning methods while that of the original model is 64. Although the configurations of pruned models are the same, the pruning schemes are different. Obviously, the errors of pruned models are different, and EMOFP always obtains much less errors than other methods. After we fine-tune these pruned models with same training strategies, as all the methods can obtain acceptable final models with similar or little larger errors compared with the original model. With the increasing compression ratio, the error is increased whether it is the pruned model or fine-tuned model. For EMOFP, the fine-tuned models are better than the original model, except in the case where CR is 7.11. Even when the pruned model only has nine filters, the error after fine-tuning is 0.0136, which is slightly larger than 0.0122. Moreover, the FLOPs of the pruned model is significantly less than that of the original model, and the FLOPs of the last pruned model are only 14% of that of original model. Thus, for the designed Conv1, EMOFP generally has a good performance for obtaining a lightweight model.

Secondly, Figure 3b shows the Pareto front of EMFP on Conv2. From the figure, we can deduce that EMFP obtains a series of uniform trade-off solutions, and their errors are acceptable. The remained filter ratios of these solutions are in the range of [0.15 0.5] which satisfy the setting of parameters $\delta_{1}$ and $\delta_{2}$, and their maximum error is approximately 0.25—which is acceptable and can become very small by fine-tuning. More detailed results on Conv2 are presented in Table 4. The difference with the results of Conv1 is that the comparison methods consist of $l_{1}$-global, $l_{1}$-layer, $l_{2}$-global and $l_{2}$-layer. From the table, we can deduce that the configuration and error of the original model are (32, 64) and 0.0083, respectively, and all the methods prune the model with three different filter compression ratios: 2.04, 3.31 and 5.65. Although their CRs are the same, the detailed pruning schemes are different, especially for different pruning ratio assignment strategies. In terms of the error of the pruned models, the EMOFP can obtain better results than all comparison methods, except in the case where filter pruning ratio is 2.04, in which case the results of our method become better and better than those of others with an increasing pruning ratio. In terms of the error of the fine-tuned model, the results of all methods are similar and approximate the error of the original model. Moreover, the fine-tuned error of EMOFP is better than the original one, except for the third pruning scheme, and EMOFP performs better than comparison methods in most cases.

Thirdly, the comparison results and the Pareto front are shown in Table 5 and Figure 3c. The Pareto front of EMFP on Conv3 is not very smooth but uniformly distributed. From the figure, we can deduce that the remaining filter ratio is in the range of [0.24 0.42] and their error is in the range [0.1 0.6]. Generally, the results of EMFP are not bad; however, the range of the pruning ratio is a little small, especially for high pruning ratio solutions. From Table 5, it is well known that EMOFP is always better than the four comparison methods regardless of the pruning scheme. The configuration of the original model is (16, 32, 64), which has 112 filters in total, and the filter’s number of pruned models is approximately 45, 34 and 27, respectively. It is worth noting that the pruned configuration will be 46, 34 and 28 when the pruning ratio assignment is layer-wise. The error of the original model is 0.0071, and the error of the final models of all methods are worse than that of original model, although our method performed better than the comparison methods. However, the errors of the final models are perfectly acceptable, even in terms of the filter compression ratio which is maximum 4.15, and the maximum error of our pruned model is 0.0104. When comparing the error of the pruned model which is not fine-tuned, the error of EMOFP is obviously smaller than that of the comparison methods, which reveals that EMOFP is better than the comparison methods on Conv3. When we focus on the FLOPs of the pruned model, EMOFP certainly obtains a lightweight model which only has approximately 20% of the FLOPs of the original model.

Finally, we present comparison results and Pareto front on Conv4 in Table 6 and Figure 3d, respectively. In Figure 3d, the Pareto front of EMFP on Conv4 is not very good the because the front is not smooth enough and the range of remaining filter ratio is not wide enough. The smallest pruned model has kept over 30% filters, and the error of this model is approximately 0.8 before fine-tuning. Meanwhile, the biggest pruned model has kept approximately 55% filters, and the error of this model is approximately 0.1. Detailed comparison results are in Table 6. It is well known that the original model has 176 filters in total with the configuration of (16, 32, 64, 64), and its error is 0.0065. Although the detailed configuration of pruned models with different methods is different, the filter’s number of each model with the same pruning ratio is similar, and they are approximately 97, 80 and 60, respectively. Moreover, the filter compression ratios of our method are 1.81, 2.2 and 2.93, respectively. Compared to the original model, the performances of all fine-tuned models are worse than that of the original model, although the their error is also acceptable, especially for models of EMOFP, where the biggest error of our fine-tuned model is only 0.0093. In terms of the error of the pruned model, EMOFP performs much better than the comparison methods. For the second pruning ratio, the error of EMOFP is 0.1867 while the best result of the comparison methods is 0.4856. Obviously, EMOFP can prune over 70% filters of Conv4 with little performance loss, and perform better than comparison methods in general. Moreover, the average FLOPs of the pruned models are approximately 20% those of the original model, which reveals that EMOFP can obtain a lightweight model with acceptable performance.

From these results on four designed models, we can make sure that EMOFP provides a series of efficient different trade-off solutions and has better performance than the comparison methods. Moreover, we can also know that with the depth of increasing depth of the model, the pruning performance decreases. For four Pareto fronts, the Pareto fronts of Conv1 and Conv2 are better than those of Conv3 and Conv4. It is well known that the filter pruning problem dimension is increasing while the model becomes increasingly deep. Therefore, the difficulty of the pruning problem is increased. For example, the pruning problem dimensions of four designed models are 64, 96, 112 and 176. Furthermore, the number of filters of each layer is also a restricted condition for optimization, which will be complex due to the increasing number of layers. It is therefore increasingly difficult to find solutions with a large filter pruning ratio, and the results show that the biggest filter compression ratio decreases when the model becomes complex. Moreover, the FLOPs of our pruned models are less obvious than those of the original model, although the FLOPs of EMOFP are not the most competitive. This is because our EMOFP is only optimized for the number of filters and does not take FLOPs into account. Generally, EMOFP surely obtains a lightweight model with acceptable performance.

#### 4.3.2. Results on LeNet

**Figure 4:**
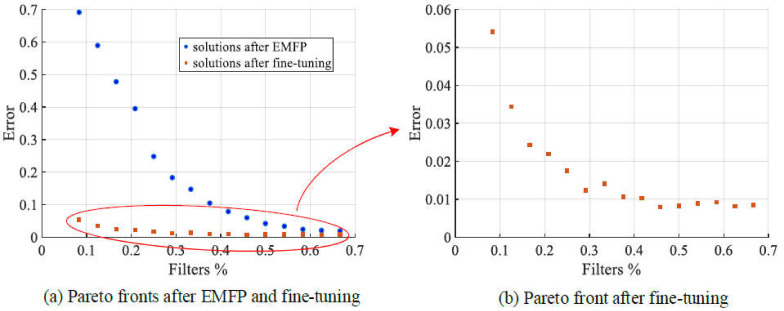
Pareto front of EMFP and scatter plot of fine-tuned solutions on LeNet.

In this part, we will show the experimental results on LeNet, which is one of the most familiar convolutional neural networks. Firstly, we plot the Pareto front of EMFP and the fitness of the fine-tuned models corresponding to Pareto solutions in Figure 4. In Figure 4a, the blue circle dot means the solution of EMFP and the red square dot denotes the solution after fine-tuning. It is well known that EMFP can obtain a very good Pareto front for which the ranges of the remained filter ratio and error are both in [0, 0.7] and the Pareto front is smooth and uniformly distributed. Moreover, as the errors of models after fine-tuning are all below 0.1, it is difficult to observe the change in these solutions in Figure 4a. In order to show them to be more precise, we plot a separate scatter figure in Figure 4b. From Figure 4b, we can know that the distribution of fine-tuned solutions is approximate to Pareto distribution. The maximum error of obtained fine-tuned models is approximately 0.054 with the remaining filter ratio of 0.08. Furthermore, the minimum error is approximately 0.0085 while the remained filter ratio is approximately 0.667.

The comparison results on LeNet are presented in Table 7. The configuration and error of the used LeNet are (8, 16) and 0.0095, respectively. For EMOFP and the comparison filter pruning methods, the minimum filter compression ratio is 1.5 when the number of remaining filters is 16, and the maximum filter compression ratio is 12 when only two filters remain. For global pruning ratio assignment methods, the $l_{1}$-layer and $l_{2}$-layer, they cannot generate a normal convolutional neural network because there is no filter in the second convolutional layer. In terms of the error of the pruned model which is not fine-tuned, EMOFP is much better than all comparison methods, especially with the increasing filter pruning ratio. Moreover, in terms of the error of the fine-tuned model, EMOFP is also better than all comparison methods, where the errors of three different pruning schemes of EMOFP are 0.0085, 0.0106 and 0.0541, respectively. It is well known that EMOFP obtained a series of valuable different trade-off pruning solutions, and that their FLOPs of are greatly less than that of the original model.

#### 4.3.3. Results on AlexNet

**Figure 5:**
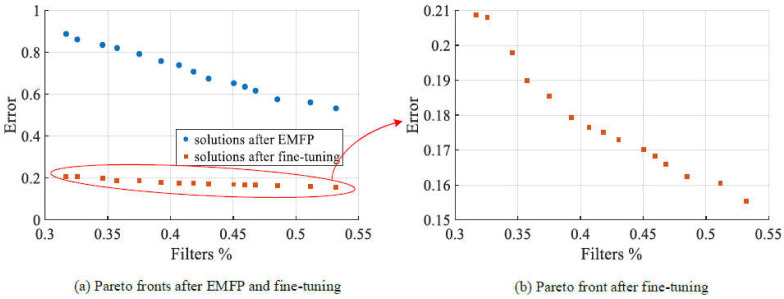
Pareto front of EMFP and scatter plot of fine-tuned solutions on AlexNet.

AlexNet was the deepest convolutional neural network used to examine the performance of EMOFP in the experimental studies. Furthermore, the detailed experimental results are shown in Figure 5 and Table 8. Firstly, we plot the Pareto front of EMFP and scatter plot of fine-tuned models in Figure 5. In Figure 5a, the blue circle dot means the solution of EMFP and the red square dot denotes the solution after fine-tuning. The Pareto front of EMFP is approximate to a line, and the errors of Pareto solutions are not small where all of them are greater than 0.5. Moreover, the range of the remaining filter ratio is [0.1 0.55], which is a little narrow. From the Pareto front, we can deduce that our EMFP can provide a series of trade-off pruned models but it suffers some difficulties of higher dimension optimization. In order to analyze the final performance of these models, we also scatter plot the fine-tuned model in Figure 5a and separately show it in Figure 5b. It is well known that the distribution of fine-tuned solutions is approximate to Pareto distribution, and the error of a fine-tuned model is in range of 0.15–0.21. All of them are worse than the original AlexNet. In total, EMOFP does not perform as well on AlexNet as it did before from Figure 5.

We present a detailed comparison of the results on AlexNet in Table 8. The filter configuration of the original AlexNet is (24, 64, 96, 96, 64) and the number of filters is 344 in total. Furthermore, the error of the original AlexNet is 0.0996. The comparison methods consist of norm-based filter pruning [13], average percentage of zeros (APoZ) [15], soft filter pruning (SFP) [16] and ThiNet [17], where APoZ and SFP are implemented on one-shot pruning framework and ThiNet belongs to iterative pruning. From Table 8, under the condition of a similar pruned model (pruning approximately 60% filters), the performance of EMOFP is not bad but just worse than SFP and ThiNet. Specifically, the configuration of the pruned model with EMOFP is (9, 20, 39, 43, 24), while that of most of comparison methods is (10, 25, 38, 38, 25), because these comparisons use the same layer pruning ratio. Norm-based filter pruning methods are obviously worse than others due to the rough filter importance estimation. SFP pruning filters, while training the model, could update the weights in time. ThiNet applies an iterative pruning framework which usually works better. For a one-shot pruning method, EMOFP achieves the error of 0.1794 on AlexNet, which is acceptable. Moreover, the FLOPs of our pruned model are only half those of the original model, because there are a lot of FLOPs in fully connected layers which are not pruned.

### 4.4. Fine-Tuning with Shared Weights or Randomly Initial Weights

From the experimental results, we observed that the performances of fine-tuned models have some differences although they have the same model structure. The difference between these pruned models before fine-tuning is their weights. Therefore, in this part, we explore the influence of different weights on the pruned model to analyze that the purpose of pruning is searching a suitable lightweight model structure or a pre-trained model which only needs to be fine-tuned. In this experiment, we rebuilt the model with random initialized weight parameters according to the configuration of the pruned model. The experimental results are shown in Table 9.

From Table 9, there are two models, Conv2 and Conv3, used to examine the difference between the two different ways of giving the weights of a pruned model. For each model, we select 10 different pruning solutions and sort them with the error of the pruned model without fine-tuning ascending. Comparing Error rf and Error sf, which are the errors of the fine-tuned model with random initialized weights and shared original weights, respectively, we can know that Error sf is always less than Error rf for all pruned models, whether it is Conv1 and Conv2. This reveals that sharing original weights is better than random initialized weights. Therefore, our pruning method not only searches for a suitable lightweight model structure but also provides suitable initial weights of the lightweight model, therefore simple fine-tuning can obtain a lightweight and well-performing model.

### 4.5. Practical Example of Cat and Dog Classification

In order to exam the performance of the proposed method, we provided a simple practical example about cat and dog classification. Firstly, we randomly selected 1000 cat images and 1000 dog images as training samples, and each RGB image with the shape of 150×150×3. Moreover, the test dataset consists of 500 cat images and 500 dog images with the same shape of the training sample. Secondly, we designed a deep convolutional neural network as a cat and dog classifier with four convolutional layers and two fully connected layers, in which the filter size was 3×3 and the number of neurons in the fully connected layers was 512 and 1. A detailed configuration and pruning results are shown in Table 10. From the results of EMOFP for cat and dog classification, it is well known that the proposed method can potentially enhance the previous model. For example, seven of the nine pruned models have better accuracy than the original classifier and the configuration of all nine pruned models is simpler.

## 5. Conclusions and Future Works

In this paper, we proposed an evolutionary multi-objective one-shot filter pruning method to design a lightweight convolutional neural network. Firstly, EMOFP is no longer an iterative pruning method and only needs to perform pruning and fine-tuning once. Secondly, a multi-objective filter pruning problem was established, which consists of two objective functions and represents the filter pruning ratio and the accuracy of the pruned model. Thirdly, we used a NSGA-II-based evolutionary multi-objective algorithm to solve the above filter pruning problem and obtain a series of different trade-off pruning solutions. Finally, *K*-pruned models were uniformly selected for fine-tuning to promote their performance. Therefore, EMOFP provides a series of different trade-off pruned models instead of a pruned model. Experimental studies of four designed models and two famous models demonstrated that our method can efficiently prune filters to obtain a lightweight convolutional neural network. Compared with the $l_{1}$-norm and $l_{2}$-norm-based one-shot filter pruning methods, EMOFP performs better than these comparison methods most of the time, especially for comparing the error of pruned model before fine-tuning, as our method is always much better than all comparison methods. Moreover, we discussed whether the purpose of pruning is to obtain a lightweight network structure or a lightweight network with shared weights. By comparing the model performance of random initial weights and shared weights, shared weights always lead to better performance. Therefore, model pruning not only searches a suitable lightweight model structure but also provides suitable initial weights of the pruned model.

There are still some unresolved issues in this paper: for example, the pruning performance of a larger model is decreased because of the increasing difficulty in optimization. Therefore, in future work, we want to explore more efficient optimization strategies for very deep convolutional neural network filter pruning.

## Figures and Tables

**Table 1:** Detailed neural network variants, consisting of the configuration, accuracy and used dataset.

Model	Conv1	Conv2	Conv3	Conv4	LeNet	AlexNet
**Dataset**	MNIST	MNIST	MNIST	MNIST	MNIST	CIFAR10
**Accuracy**	0.9878	0.9917	0.9929	0.9935	0.9905	0.9004
**Configuration**	64@(3,3)	32@(3,3)	16@(3,3)	16@(3,3)	8@(5,5)	24@(3,3)
	(,128)	64@(3,3)	32@(3,3)	32@(3,3)	16@(5,5)	64@(5,5)
	(128,10)	(,128)	64@(3,3)	64@(3,3)	(,120)	96@(3,3)
		(128,10)	(,128)	64@(3,3)	(120,84)	96@(3,3)
			(128,10)	(,128)	(84,10)	64@(5,5)
				(128,10)		(,1024)
						(1024,1024)
						(1024,10)

**Table 2:** Detailed parameters of the evolutionary multi-objective one-shot filter pruning.

Parameter	Meaning	Value
*N*	The number of individuals in population	50
*G*	The maximum of generations	200
pc	The probability of crossover	0.9
pm	The probability of mutation	0.2
$\delta_{1}$	The lower bound of error in Equation (7)	{0.01, 0.5}
$\delta_{2}$	The upper bound of error in Equation (7)	{0.7, 0.9}

**Table 3:** Pruning results of Conv1 on MNIST. Error p and Error f mean the error of the pruned model before fine-tuning and the fine-tuned model, respectively. CR means the filter compression ratio of the pruned model.

Model	Method	Config	Remained Filter %	Error p	Error f	Relative Error %	CR	FLOPs
Conv1	original	(64)	-	-	0.0122	-	-	3.21 M
	$l_{1}$	(18)	28.13	0.1846	0.0123	0.82	3.55	0.91 M
		(13)	20.31	0.2984	0.0121	−0.82	4.92	0.66 M
		(9)	14.06	0.4188	0.0137	12.30	7.11	0.45 M
	$l_{2}$	(18)	28.13	0.1823	0.012	−1.64	3.55	0.91 M
		(13)	20.31	0.3355	0.0122	0	4.92	0.66 M
		(9)	14.06	0.4716	0.0136	11.48	7.11	0.45 M
	EMOFP	(18)	28.13	0.05	0.0118	−32.79	3.55	0.91 M
		(13)	20.31	0.0523	0.012	−1.64	4.92	0.66 M
		(9)	14.06	0.156	0.0136	11.48	7.11	0.45 M

**Table 4:** Pruning results of Conv2 on MNIST.

Model	Method	Config	Remained Filter %	Error p	Error f	Relative Error %	CR	FLOPs
Conv2	original	(32, 64)	-	-	0.0083	-	-	842.82 K
	$l_{1}$-global	(20, 27)	48.96	0.0634	0.0081	−0.85	2.04	351.33 K
		(15, 14)	30.21	0.4339	0.0095	14.46	3.31	182.23 K
		(9, 8)	17.71	0.5497	0.0097	16.87	5.65	104.38 K
	$l_{1}$-layer	(16, 31)	48.96	0.1842	0.0074	−10.84	2.04	400.65 K
		(10, 19)	30.21	0.5242	0.008	−3.61	3.31	244.50 K
		(6, 12)	17.71	0.5692	0.0099	19.27	5.65	154.50 K
	$l_{2}$-global	(20, 27)	48.96	0.0933	0.008	−3.61	2.04	351.33 K
		(15, 14)	30.21	0.2454	0.0086	3.61	3.31	182.23 K
		(8, 9)	17.71	0.632	0.0091	9.64	5.65	116.90 K
	$l_{2}$-layer	(16, 31)	48.96	0.1841	0.0075	−9.64	2.04	400.65 K
		(10, 19)	30.21	0.2291	0.0074	−10.84	3.31	244.50 K
		(6, 12)	17.71	0.6058	0.0101	21.69	5.65	154.50 K
	EMOFP	(12, 35)	48.96	0.1001	0.0073	−12.05	2.04	449.38 K
		(10, 19)	30.21	0.1005	0.0077	−7.23	3.31	244.50 K
		(8, 9)	17.71	0.2491	0.0098	18.07	5.65	116.90 K

**Table 5:** Pruning results of Conv3 on MNIST.

Model	Method	Config	Remained Filter %	Error p	Error f	Relative Error %	CR	FLOPs
Conv3	original	(16, 32, 64)	-	-	0.0071	-	-	196.39 K
	$l_{1}$-global	(16, 17, 12)	40.18	0.3622	0.0086	21.13	2.49	39.07 K
		(16, 15, 3)	30.36	0.677	0.0168	136.62	3.29	14.90 K
		(15, 11, 1)	24.11	0.8716	0.0407	473.24	4.15	8.31 K
	$l_{1}$-layer	(7, 13, 26)	41.07	0.4395	0.0086	21.13	2.43	70.32 K
		(5, 10, 19)	30.36	0.5054	0.0105	47.89	3.29	50.75 K
		(4, 8, 16)	25	0.7272	0.0109	53.52	4	42.38 K
	$l_{2}$-global	(16, 17, 12)	40.18	0.2142	0.0087	22.54	2.49	39.07 K
		(16, 12, 6)	30.36	0.4856	0.0137	92.96	3.29	21.43 K
		(15, 10, 2)	24.11	0.7025	0.0205	188.73	4.15	10.51 K
	$l_{2}$-layer	(7, 13, 26)	41.07	0.5695	0.0084	19.31	2.43	70.32 K
		(5, 10, 19)	30.36	0.62	0.0111	56.34	3.29	50.75 K
		(4, 8, 16)	25	0.615	0.0113	59.15	4	42.38 K
	EMOFP	(8, 13, 24)	40.18	0.1014	0.008	12.68	2.49	65.75 K
		(7, 10, 17)	30.36	0.1867	0.0084	18.31	3.29	46.18 K
		(3, 9, 15)	24.11	0.5989	0.0104	46.48	4.15	40.10 K

**Table 6:** Pruning results of Conv4 on MNIST.

Model	Method	Config	Remained Filter %	Error p	Error f	Relative Error %	CR	FLOPs
Conv4	original	(16, 32, 64, 64)	-	-	0.0065	-	-	139.05 K
	$l_{1}$-global	(16, 30, 37, 14)	55.11	0.5102	0.0079	21.54	1.81	44.38 K
		(16, 30, 27, 7)	45.45	0.7952	0.0109	67.69	2.2	31.27 K
		(16, 26, 15, 3)	34.09	0.9152	0.0276	324.62	2.93	18.94 K
	$l_{1}$-layer	(9, 18, 35, 35)	55.11	0.4502	0.008	23.08	1.81	48.00 K
		(7, 15, 29, 29)	45.45	0.5451	0.009	38.46	2.2	34.98 K
		(6, 11, 22, 22)	34.66	0.7669	0.0252	287.69	2.89	22.56 K
	$l_{2}$-global	(16, 32, 38, 11)	55.11	0.6379	0.0099	52.31	1.81	44.30 K
		(16, 29, 30, 5)	45.45	0.6235	0.013	1	2.2	30.85 K
		(16, 24, 19, 1)	34.09	0.9066	0.2965	4461.54	2.93	18.57 K
	$l_{2}$-layer	(9, 18, 35, 35)	55.11	0.4294	0.0081	24.62	1.81	48.00 K
		(7, 15, 29, 29)	45.45	0.577	0.0079	21.54	2.2	34.98 K
		(6, 11, 22, 22)	34.66	0.7596	0.0108	66.15	2.89	22.56 K
	EMOFP	(8, 19, 34, 36)	55.11	0.1086	0.0082	26.15	1.81	48.32 K
		(9, 13, 28, 30)	45.45	0.1793	0.0086	32.31	2.2	34.19 K
		(5, 9, 24, 22)	34.09	0.6199	0.0093	43.08	2.93	22.49 K

**Table 7:** Pruning results of LeNet on MNIST.

Model	Method	Config	Remained Filter %	Error p	Error f	Relative Error %	CR	FLOPs
LeNet	original	(8, 16)	-	-	0.0095	-	-	90.09 K
	$l_{1}$-global	(7, 9)	66.67	0.0585	0.0091	−4.21	1.5	59.91 K
		(7, 2)	37.5	0.4898	0.0171	80	2.67	30.58 K
		(2, 0)	8.33	-	-	-	-	-
	$l_{1}$-layer	(5, 11)	66.67	0.0464	0.0091	−4.21	1.5	67.09 K
		(3, 6)	37.5	0.5573	0.0141	48.42	2.67	45.94 K
		(1, 1)	8.33	0.8472	0.0585	515.79	12	25.79 K
	$l_{2}$-global	(7, 9)	66.67	0.0325	0.0086	−9.47	1.5	59.91 K
		(6, 3)	37.5	0.4698	0.02	110.53	2.67	34.57 K
		(2, 0)	8.33	-	-	-	-	-
	$l_{2}$-layer	(5, 11)	66.67	0.0486	0.0089	−6.32	1.5	67.09 K
		(3, 6)	37.5	0.4066	0.0121	27.37	2.67	45.94 K
		(1, 1)	8.33	0.8565	0.0722	660	12	25.79 K
	EMOFP	(6, 10)	66.67	0.02	0.0085	−10.53	1.5	63.55 K
		(4, 5)	37.5	0.1054	0.0106	11.58	2.67	42.25 K
		(1, 1)	8.33	0.6915	0.0541	469.47	12	25.79 K

**Table 8:** Comparison of the pruning results of AlexNet on CIFAR10.

Model	Method	Config	Remained Filter %	Error	Relative Error %	CR	FLOPs
AlexNet	original	(24, 64, 96, 96, 64)	-	0.0996	-	-	11.67 M
	l1-global [13]	(24, 53, 40, 14, 4)	39.24	0.252	153.01	2.55	2.93 M
	l1-layer [13]	(10, 25, 38, 38, 25)	39.53	0.186	86.75	2.53	5.58 M
	l2-global [13]	(24, 51, 42, 14, 4)	39.24	0.2232	124.10	2.55	2.92 M
	l2-layer [13]	(10, 25, 38, 38, 25)	39.53	0.1846	85.34	2.53	5.58 M
	APoZ [15]	(10, 25, 38, 38, 25)	39.53	0.1801	80.82	2.53	5.58 M
	SFP [16]	(10, 25, 38, 38, 25)	39.53	0.1735	73.90	2.53	5.58 M
	ThiNet [17]	(10, 25, 38, 38, 25)	39.53	0.1612	61.85	2.53	5.58 M
	EMOFP	(9, 20, 39, 43, 24)	39.24	0.1794	80.12	2.55	5.44 M

**Table 9:** Pruning results with different fine-tuning strategies on Conv2 and Conv3. Error p means the error of pruned model which is not fine-tuned, and Error sf and Error rf mean the error of the fine-tuned model with random initialized weight and shared original weight, respectively.

Model	Solution No.	Remained Filter %	Error p	Error rf	Error sf
Conv2	1	0.4896	0.1001	0.0101	0.0073
	2	0.3854	0.1002	0.0095	0.0079
	3	0.3125	0.1004	0.009	0.0086
	4	0.3021	0.1005	0.0103	0.0077
	5	0.2917	0.1007	0.0103	0.0081
	6	0.2708	0.101	0.0101	0.0089
	7	0.2396	0.1062	0.0097	0.0088
	8	0.2188	0.1408	0.0106	0.0106
	9	0.2083	0.1909	0.0106	0.0095
	10	0.1771	0.2491	0.0118	0.0098
Conv3	1	0.4107	0.1001	0.0104	0.01
	2	0.4018	0.1014	0.0102	0.008
	3	0.3929	0.1047	0.012	0.0097
	4	0.3839	0.1507	0.0108	0.0093
	5	0.3125	0.1634	0.0115	0.0086
	6	0.3036	0.1867	0.0096	0.0084
	7	0.2946	0.2847	0.0108	0.0088
	8	0.2857	0.3038	0.0119	0.0095
	9	0.2589	0.4949	0.0128	0.011
	10	0.2411	0.5989	0.0147	0.0104

**Table 10:** Pruning results of EMOFP for cat and dog classification. Solution No. 0 means the information of original CNN classifier.

Solution No.	Configuration of Filters	Accuracy
0	(32, 64, 128, 128)	0.8150
1	(18, 27, 65, 59)	0.8356
2	(18, 26, 54, 54)	0.8312
3	(14, 25, 41, 37)	0.8003
4	(19, 28, 45, 43)	0.8254
5	(18, 32, 70, 63)	0.8434
6	(20, 33, 55, 51)	0.8454
7	(16, 27, 32, 43)	0.8157
8	(13, 26, 35, 40)	0.8125
9	(15, 29, 45, 39)	0.8293

## Data Availability

Not applicable.
